# Binding and activation of host plasminogen on the surface of *Francisella tularensis*

**DOI:** 10.1186/1471-2180-10-76

**Published:** 2010-03-12

**Authors:** Shawn R Clinton, James E Bina, Thomas P Hatch, Michael A Whitt, Mark A Miller

**Affiliations:** 1 Department of Molecular Sciences, The University of Tennessee Health Science Center, 858 Madison Avenue, Memphis, Tennessee 38163, USA

## Abstract

**Background:**

*Francisella tularensis *(FT) is a gram-negative facultative intracellular coccobacillus and is the causal agent of a life-threatening zoonotic disease known as tularemia. Although FT preferentially infects phagocytic cells of the host, recent evidence suggests that a significant number of bacteria can be found extracellularly in the plasma fraction of the blood during active infection. This observation suggests that the interaction between FT and host plasma components may play an important role in survival and dissemination of the bacterium during the course of infection. Plasminogen (PLG) is a protein zymogen that is found in abundance in the blood of mammalian hosts. A number of both gram-positive and gram-negative bacterial pathogens have the ability to bind to PLG, giving them a survival advantage by increasing their ability to penetrate extracellular matrices and cross tissue barriers.

**Results:**

We show that PLG binds to the surface of FT and that surface-bound PLG can be activated to plasmin in the presence of tissue PLG activator *in vitro*. In addition, using Far-Western blotting assays coupled with proteomic analyses of FT outer membrane preparations, we have identified several putative PLG-binding proteins of FT.

**Conclusions:**

The ability of FT to acquire surface bound PLG that can be activated on its surface may be an important virulence mechanism that results in an increase in initial infectivity, survival, and/or dissemination of this bacterium *in vivo*.

## Background

*Francisella tularensis *(FT) is a Gram-negative intracellular pathogen that is the etiological agent of a multi-syndromic disease with a high morbidity/mortality that is referred to as tularemia. The pneumonic form of tularemia is of particular concern because of the high mortality rate (up to 60%) following inhalation of as few as ten organisms [1-4]. *Francisella *species are found throughout the Northern Hemisphere and infect a variety of vertebrate and invertebrate hosts [5,6]. Infections with FT can be contracted from blood sucking insects, such as the deer fly [5,7], mosquitoes [8,9], and ticks [5,7,10], and by open-wound contact with infected animal tissue [5,11,12].

Upon entry into a susceptible vertebrate host, FT is readily phagocytized by resident macrophages and dendritic cells and quickly escapes into the cytoplasm [13,14] where it multiplies. Late in its replicative cycle, FT induces apoptotic death of the host phagocyte, resulting in release of progeny bacteria that can infect new host cells. Recent studies have shown that significant numbers of FT are found in the acellular plasma fraction of mice infected intradermally or intranasally with either FT Live Vaccine Strain (LVS) (Type B) or FT Schu S4 (Type A) [15], and intranasally with FT *novicida *[16]. These findings suggest that, in addition to utilizing the intracellular cytoplasmic niche for replication and protection from humoral immunity, FT may also have a significant extracellular phase. Several studies have shown that deposition of host complement component C3 on the surface of FT is required for opsonophagocytosis by activating CR3 and CR4-mediated phagocytosis by macrophages and dendritic cells [14,17,18]. It is also known that FT is relatively resistant to complement-mediated lysis [19]. A recent report suggested that resistance of FT to membrane attack complex-mediated lysis may be due (at least in part) to its ability to bind to factor H from host plasma [20]. It is possible that the ability of FT to bind to factor H and potentially to other host plasma components plays a significant role in its pathogenesis.

It has been long established that a broad spectrum of both gram-positive and gram-negative bacterial pathogens gain a survival advantage by interacting with components of the host coagulation/fibrinolytic system in humans [21-24]. For instance, the ability to acquire surface-associated plasmin has been documented as an important virulence mechanism in Group A streptococci [25], *Borrelia burgdorferi *[26], and *Yersinia pestis *[27] by aiding in the organism's ability to penetrate the extracellular matrix and to disseminate to distal sites in the host. Plasminogen (PLG) is a 92-kDa glycoprotein zymogen that is involved in fibrinolysis. This precursor protein is converted to an active serine protease (plasmin) by cleavage of the peptide bond between residues R^560^and V^561 ^*in vivo *via urokinase-type (uPA) and/or tissue-type (tPA) PLG activators. Plasmin has an important role in blood clot resolution because of its role in the degradation of fibrin polymers. Because plasmin has other substrates that include pro-collagenases, pro-metalloproteinases, and extracellular matrix proteins, such as fibronectin, laminin, and vitronectin, the ability of a bacterium to acquire surface-associated plasmin can result in an enhanced ability of the pathogen to penetrate the extracellular matrix and to disseminate to distal sites in the host [21,23,25]. In this report we show that PLG binds to the surface of FT *in vitro *and that surface-bound PLG can be converted to the active plasmin form. In addition, using a combination of Far-Western blotting analyses coupled with proteomic methodologies, we have identified several FT proteins that can bind to human PLG *in vitro*.

## Results

### Binding of PLG from fresh human plasma to the surface of FTLVS

**Figure 1:**
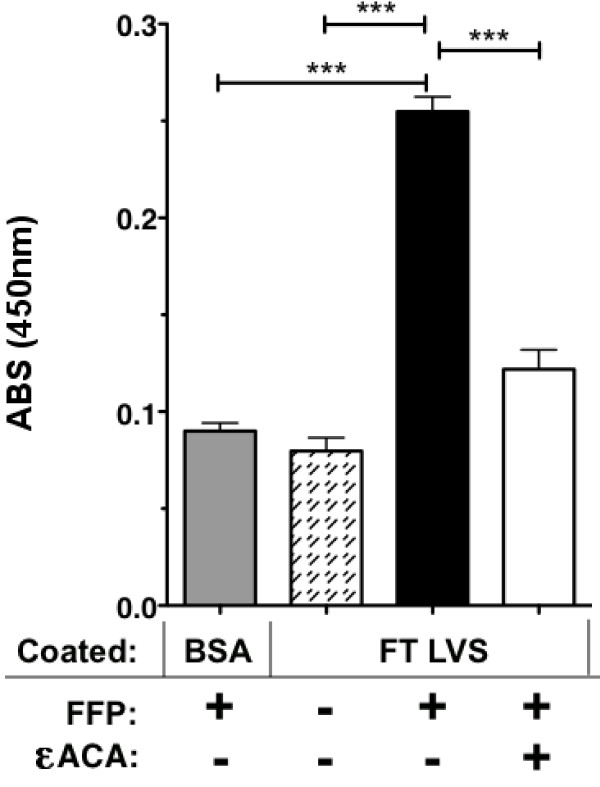
**FT binds to PLG from human plasma**. FTLVS cultured to mid-log phase in BHI broth were bound to wells of microtiter plates and then incubated for 1 hour with fresh frozen human plasma (FFP) in the presence or absence of 100 mM ε-amino caproic acid (εACA), a PLG-binding inhibitor. A modified ELISA was performed to measure FTLVS-bound PLG. The results shown are representative of 3 experiments of similar design. Bars indicate +/- SEM in triplicate. Statistical analysis was performed via one-way ANOVA using a Dunnett's Multiple Comparison post-test (*** P < .001).

**Figure 2:**
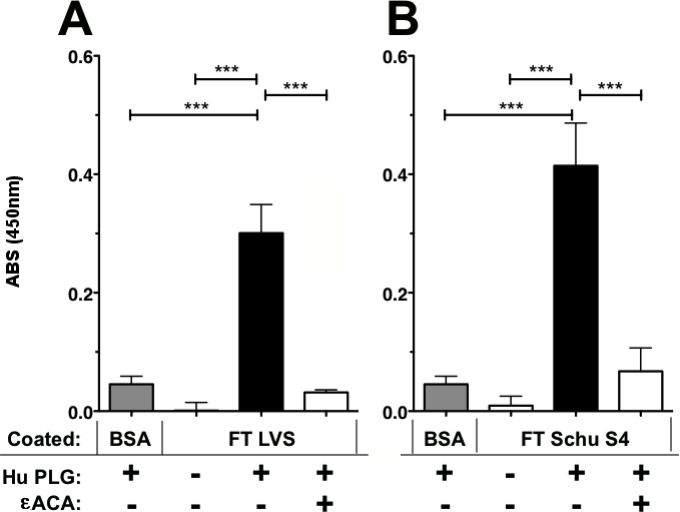
**Purified huPLG binds to FTLVS and FTSchuS4**. FTLVS (**Panel A**) and FTSchuS4 (**Panel B**) were bound to microtiter wells and incubated for 2 hours with purified huPLG (3 μg/ml) in the presence or absence of 10 mM εACA). A modified ELISA was performed to measure FTLVS-bound huPLG. The results shown are representative of four (**Panel A**) and one (**Panel B**) experiments, respectively, of similar design. Bars indicate +/- SEM in triplicate. Statistical analysis was performed via one-way ANOVA using a Dunnett's Multiple Comparison post-test (*** P < .001).

**Figure 3:**
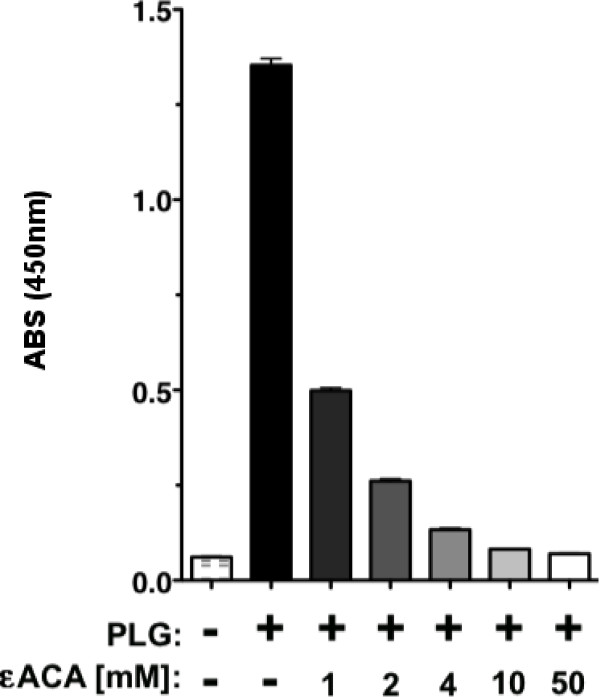
**εACA inhibits huPLG binding to FT in a dose-dependent fashion**. FTLVS was coated onto microtiter plate wells and incubated for 2 hours with purified huPLG (3 μg/mL) in the presence or absence of titrated concentrations of εACA. The results shown are representative of 3 experiments of similar design. Bars indicate +/- SEM in triplicate. Statistical analysis performed via one-way ANOVA using a Kruskal-Wallis test determined a p-value of < 0.0001.

**Figure 4:**
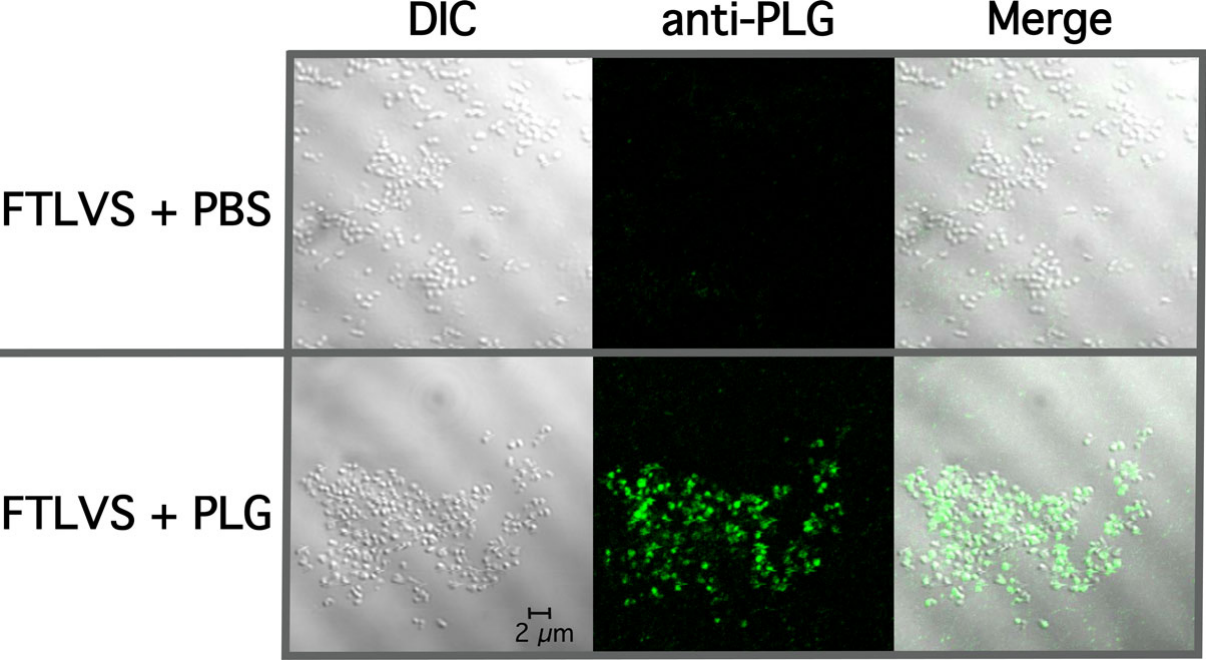
**PLG binds to the outer envelope of FT**. Laser scanning confocal microscopy of PLG-associated FTLVS was performed as described in "Materials and Methods". Bound huPLG ligand was detected using sheep anti-human PLG antibody followed by incubation with Dylight-488 conjugated donkey, anti-sheep/goat IgG secondary antibody. Samples were visualized using a Zeiss LSM 510 confocal microscope.

We used an ELISA assay to determine that PLG in fresh frozen plasma (FFP) binds to FTLVS grown to mid-log phase in BHI (Figure 1). Binding was inhibited when ε-aminocaproic acid (εACA), known to inhibit binding of PLG to lysine groups in proteins, was included in the incubation mixture. To help eliminate the possibility of non-specific binding of PLG due to its high concentrations in human plasma and also to rule out the contributions of other plasma proteins, we used purified human Glu-PLG (huPLG) and noted similar results to those observed when FFP was used (Figure 2A). We also found that huPLG binds to the highly virulent Schu S4 strain of FT at moderately higher levels than observed with FTLVS (Figure 2B). We confirmed that binding of huPLG to FT is a lysine-dependent interaction by showing that increasing concentrations of εACA can inhibit binding of huPLG to FTLVS in a dose-dependent fashion (Figure 3). When similar concentrations of glycine were used as an inhibitor control, no inhibition of huPLG binding was observed (data not shown). Confocal microscopic analyses suggested that huPLG binds to the surface of FT (Figure 4); however, it is possible that some of the staining observed was the result of huPLG penetration into the outer envelope of FT. Although is has been reported that culture media composition can have a significant impact of the surface properties and virulence characteristics of FTLVS [28], we observed no differences in the ability of PLG to bind to the surface of FTLVS grown in modified Mueller-Hinton medium vs. brain-heart infusion broth (data not shown).

### Plasmin activation on the surface of FT LVS *in vitro *by a PLG activator

**Figure 5:**
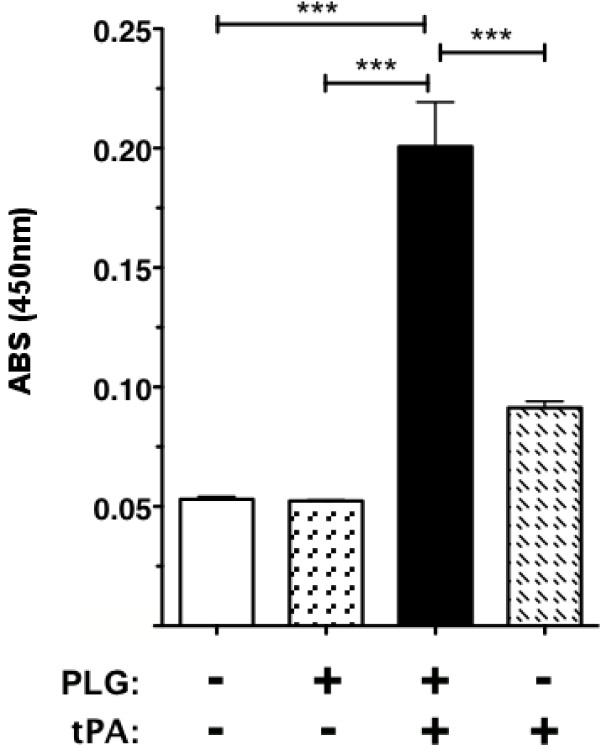
**FT surface-bound huPLG can be converted to plasmin**. FTLVS was incubated with huPLG at a concentration of 96 μg/mL. After removal of unbound huPLG, a chromogenic plasmin substrate (D-VLK-pNA), tissue PLG activator (tPA), or both were then added to test the proteolytic ability of each sample preparation. Conversion of the chromogenic substrate was measured by comparison of Δ405 nm. The results shown are representative of 3 experiments of similar design. Bars indicate +/- SEM in triplicate. Statistical analysis was performed via one-way ANOVA using a Dunnett's Multiple Comparison post-test (*** P < .001).

**Figure 6:**
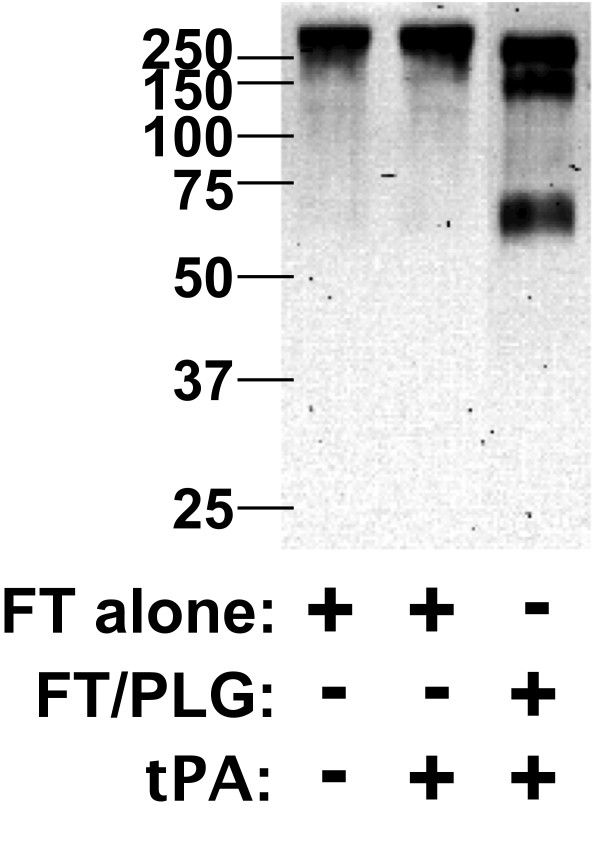
**Fibronectin is a substrate for plasmin bound to FT**. FTLVS (10^9 ^CFU) were incubated with 100 μg of huPLG and 0.5 μg tissue tPA for 1 hour at 37°C. After removal of unbound huPLG and tPA, 3 μg fibronectin was added and allowed to incubate for 24 hours at 37°C. Supernatant from each preparation were separated by SDS-PAGE and transferred to PVDF membrane. Degradation of fibronectin was detected by Western blot analysis as described in "Materials and Methods".

In other bacterial systems, surface-bound PLG can be converted to its proteolytically active plasmin form that contributes to the organism's virulence [21-24]. To test whether huPLG bound to FTLVS can be converted to plasmin, we used a chromogenic plasmin substrate (H-D-Val-Leu-Lys-pNA) to detect proteolytic activity following the addition of tissue PLG activator (tPA) (Figure 5). We also found that plasmin on the surface of FT can break down fibronectin (Figure 6), suggesting that FT-bound plasmin can potentially participate in the degradation of extracellular matrices.

### Identification of putative *Francisella *PLG-binding membrane proteins

**Figure 7:**
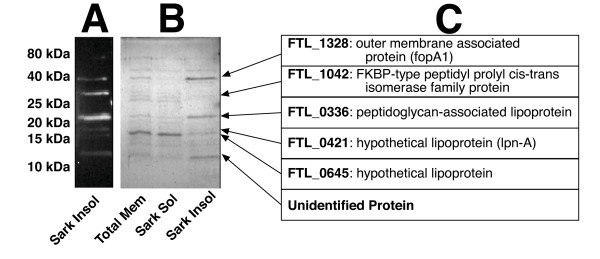
**Identification of putative PLG-binding proteins of FT**. Sarkosyl-soluble and insoluble protein fractions of FTLVS were separated by SDS-PAGE and transferred to PVDF membrane. Membranes were then blotted with huPLG (3 ug/mL) followed by anti-PLG antibody and HRP-conjugated secondary antibody to detect PLG-binding proteins (**Panel A**). Protein bands on an identical Coomassie Blue-stained SDS-PAGE gel corresponding to those identified via blotting (**Panel B**) were excised and identified using proteomic methodologies (**Panel C**).

We used Far-Western Blot methodology and mass spectrometry to identify potential PLG receptors in the Sarkosyl soluble and insoluble FTLVS membrane fractions (Figure 7). Sarkosyl is a weak anionic detergent in which many outer membrane proteins of Gram-negative bacteria are insoluble [29]. We transferred the Sarkosyl-treated proteins to a PVDF membrane and incubated the membrane with PLG and identified bound PLG by reaction with anti-PLG mAbs (Figure 7a). We used the relative migration rates of the reactive bands to identify the reactive proteins on a duplicate Coomassie-stained polyacrylamide gel (Figure 7b), which were then excised for proteomic analysis by mass spectrometry. Several prominent PLG-binding proteins were noted in the total membrane fraction of FTLVS, all but one of which was found in the Sarkosyl insoluble fraction (Figure 7b). The identity of the prominent proteins from this assay (Figure 7c) are the products of the following genes: FTL_1328 (outer membrane associated protein, fopA1), FTL_1042 (FKBP-type peptidyl-prolyl cis-trans isomerase family protein), FTL_0336 (peptidoglycan-associated lipoprotein), FTL_0421 (hypothetical lipoprotein, lpn-A), and FTL_0645 (hypothetical lipoprotein).

## Discussion

Until recently FT has been considered an intracellular pathogen whose dissemination to tissues distal to the site of initial infection was highly dependent on its ability survive within host macrophages. The observation that FT can be found in relatively high numbers in the acellular plasma fraction of its mammalian host [15,16] suggested that FT may have a significant extracellular component to its life cycle and that interactions between FT and one or more plasma proteins could contribute to its ability to disseminate within the host. There are a number of examples of bacterial pathogens that utilize interactions with host plasma components to enhance their ability to colonize and to penetrate the extracellular matrices of host cells/tissues. A wide range of bacterial pathogens (including *Francisella*) subvert the destructive mechanisms of the complement cascade by acquiring surface-bound complement control proteins [20,30-34]. Moreover, a number of Gram-positive bacterial pathogens including *streptococcal *spp. [35,36], *staphylococcal *spp. [37-40], and *Bacillus anthracis *[41,42], as well as Gram-negative bacteria such as *Pseudomonas aeruginosa *[43] have been shown to augment their invasive capacity by interacting with fibrinogen, fibronectin, and/or PLG. *Yersinia pestis *is probably the best-characterized example of a pathogen that exploits the host fibrinolytic system to penetrate host tissues. *Yersinia *expresses a surface serine protease (designated Pla) whose substrates include several complement components, PLG, and alpha2-antiplasmin (the primary circulating inhibitor of plasmin). Pla also has adhesin activity and binds to laminin (a glycoprotein of mammalian basement membranes). Because Pla upregulates plasmin activity, and because laminin is a substrate of plasmin, *Yersinia *can very efficiently penetrate basement membranes of host tissues [for review, see Suomalainen et. al. [44]]. Clearly, interaction with plasma components is a strategy that is used by many bacterial pathogens to gain a survival advantage within their hosts.

The goal of the studies described here was to determine whether FT has the potential to use the host fibrinolytic system (specifically PLG) to enhance its ability to penetrate/disseminate following infection of a mammalian host. Our results indicate that both FTLVS and FTSchuS4 are able to acquire surface bound PLG *in vitro *and that this zymogen can be converted by a host-derived PLG activator into its active serine protease form (plasmin) while bound to FTLVS. The ability of PLG to bind its ligands typically involves its lysine-binding kringle domains. This specific interaction between PLG and exposed lysine residues can be inhibited with the lysine-analogue εACA and, to a lesser extent, with free lysine. Our findings revealed that binding of PLG to the surface of FTLVS could be inhibited by εACA in a dose-dependent fashion. Moreover, we showed that plasmin bound to the surface of FT could degrade fibronectin. This finding supports our hypothesis that the ability of FT to bind to serum plasmin may enhance its ability to penetrate extracellular matrices, enhancing its ability to disseminate *in vivo*.

Using a ligand-blotting technique coupled with proteomic methodologies we identified five FTLVS proteins that were able to bind to PLG, each of which are highly conserved among the various FT type A and B strains. Three of these proteins are lipoproteins (gene products of FTL_0336, FTL_0421, and FTL_0645). Two of the lipoproteins are unique to FT, while the third, peptidoglycan-associated lipoprotein (PAL), is highly conserved among gram-negative bacteria. The specific use of surface-exposed lipoproteins as receptors for host PLG is not unusual and has been well documented in other human bacterial pathogens, such as some members of the genus *Borrelia *and *Treponema*. Several members of the genus *Borrelia *use complement regulator-acquiring surface proteins (CRASP) to bind both PLG and complement factor H to aid in the ability of the organism to both disseminate and to resist innate immunity [45-50]. An additional example of a PLG-binding lipoprotein is OppA of *Treponema denticola*, which has been suggested to play a role in periodontal disease in humans [51]. With this in mind, there lies the possibility that lipoproteins of *Francisella *species may have the capacity to bind multiple host-derived proteins in addition to PLG.

Here we have shown that FT can bind to PLG and that surface-bound PLG can be activated by tPA to its proteolytic form (plasmin). The binding of PLG on the surface of FT could play a role in several phases of tularemia, including the initial entry into the host through insect bites and/or broken skin where active fibrinolytic processes would provide an early opportunity for FT to acquire proteolytic activity that might augment the establishment or dissemination of infection. During later phases of tularemia the acquisition of plasmin on the cell surface may contribute to its pathogenicity by degrading host innate effector molecules and extracellular matrix components. Based on the new report that FT-bound plasmin can degrade immunoglobulins [52], as well as the established ability of FT to acquire surface-bound factor H [20], it also appears likely that FT uses plasma components to interfere with host humoral immune mechanisms throughout the course of FT infection. Future studies to identify additional plasma components that can be surface acquired by FT may uncover additional virulence mechanisms used by this pathogen during its extracellular life cycle.

## Conclusions

FT interacts with at least two serum components (plasmin, and complement factor H), and it seems likely that FT also uses interactions with additional host serum components to gain a survival advantage. Our lab is examining FT interactions with additional targets, including fibrinogen and fibronectin, both of which are substrates for plasmin and are host components that are known to be exploited by numerous pathogens for adhesion to and penetration of extracellular matrix layers. The interaction of FT with host serum components may play a significant role in the survival and dissemination of this highly pathogenic bacterium. Gaining a better understanding of these interactions could be a critical step in the development of therapeutic and prophylactic interventions for tularemic disease.

## Methods

### Bacterial strains and culture

*F. tularensis *Live Vaccine Strain (FTLVS) was a kind gift of Dr. Karen Elkins (FDA, Bethesda, MD). FT Schu S4 was obtained from the CDC. All bacterial cultures were grown overnight in Brain-Heart Infusion broth (37 g/L, pH 6.8) from frozen stocks at 37°C with shaking to mid-log phase (OD_600 _= ~0.7) before use.

### Reagents

Human fresh frozen plasma (FFP) was purchased from Lifeblood Mid-South Regional Blood Center (Memphis, TN). Purified human Glu-PLG (huPLG), human single-chain tissue PLG activator (tPA), and the plasmin colorimetric substrate (H-D-Val-Leu-Lys-pNA) were purchased from Molecular Innovations (Novi, MI). Bovine serum albumin (fraction V) was purchased from Thermo-Fisher Scientific (Pittsburgh, PA). Polyclonal sheep anti-human PLG, anti-human fibronectin, and donkey anti-sheep/goat IgG:Dylight-488 antibody preparations purchased from AbD Serotec (Raleigh, NC). Monoclonal anti-goat/sheep IgG-horseradish peroxidase conjugated secondary antibody (clone GT-34) and ε-aminocaproic acid (A7824) were purchased from Sigma-Aldrich (St. Louis, MO). Ninety-six well MAXISORP ELISA plates were purchased from Nunc (Rochester, NY).

### PLG binding ELISA assays

FTLVS was cultured overnight to mid-log phase, pelleted at 6,400 × g for 30 minutes, washed twice with phosphate-buffered saline (PBS), and resuspended in PBS with 0.1% Na azide to an OD_600 _= 0.1. The resulting bacterial suspension was added to microtiter plates (100 μL/well; approximately 2.5 × 10^8 ^bacterial cells) before being incubated overnight at 4°C to facilitate binding. The wells were then washed twice with 200 μL of Tris-buffered saline (TBS) pH 7.45 containing 0.05% Tween-20 (TBST) to remove unbound bacteria and then pre-blocked with 200 μL of TBST containing 1% bovine serum albumin (1% BSA-TBST) for 1 hour at RT° to prevent non-specific protein binding. After removal of the blocking solution, 90% citrated human plasma or 3 μg/mL huPLG in 1% BSA-TBST was added to each well (100 μL), with or without the indicated concentrations of ε-amino caproic acid (εACA), and incubated for 1-2 hours at 37°C with gentle rocking. Wells were washed three times with TBST and then sheep anti-human PLG-specific antibody (1:2,000 dilution in 1% BSA-TBST) was added (100 μL/well) and allowed to incubate for 1 hour at 37°C. Unbound primary antibodies were removed by washing three times with TBST, followed by the addition of HRP-conjugated anti-sheep/goat IgG mAb (GT-34, 1:5,000 dilution in 1% BSA-TBST; 100 μL/well) and incubation for 1 hour at 37°C. Unbound secondary antibodies were removed by washing four times with TBST, and OptEIA TMB colorimetric substrate solution (Becton-Dickenson, Franklin Lakes, NJ) was added to each well (100 μL/well) and incubated at 37°C for 20 min. to allow color development. Absorbance at 450 nm was determined using a SpectraMAX 340 plate reader (Molecular Devices, Sunnyvale, CA).

### Indirect immunofluorescence assays

FTLVS was cultured and washed as described above. After diluting the washed bacteria to OD_600 _= 0.1, 1 mL aliquots were incubated with a total of 40 μgs of PLG or PBS (negative control) for 30 minutes at 37°C with gentle rotation. Bacteria were then washed three times with PBS by centrifugation, resuspended in 100 μL of PBS, followed by spotting 20 μL of each sample onto glass coverslips. The samples were then air-dried overnight at 37°C. After methanol fixation, the coverslips were blocked with 1% BSA-PBS at room temperature before adding sheep anti-human PLG (1:100 diluted in 1% BSA-PBS) for 30 minutes at room temperature. The coverslips were gently washed with PBS before adding donkey anti-sheep/goat IgG:Dylight-488 (1:100 diluted in 1% BSA-PBS), followed by incubation for 30 minutes at room temperature. After washing again with PBS, coverslips were mounted onto glass slides using 100% glycerol containing 0.1 M n-propyl gallate and images were collected on a Zeiss LSM 510 confocal microscope with an Axiovert 100 M base with a 100× Plan Apochromat 1.4 NA oil DIC objective using the argon laser for 488 nm excitation and 505-530 nm bandpass emission filter for imaging Dylight488 fluorescence and the HeNe1 543 nm laser for illumination of the DIC images. Both images were collected using identical detector gain and amplifier offset settings, and the images shown are 1.0 μm optical slices. Digital images were visualized using Zeiss AxioVision LE software.

### Chromogenic plasmin activation assay

FTLVS was cultured overnight to mid-log phase, washed twice with TBS and then resuspended in TBS to an OD_600 _of 0.7. Aliquots of the bacterial suspension (50 μL) was added to 50 μL of TBS alone or TBS containing huPLG (192 μg/mL) and incubated for 1 hour at 37°C. The cells were washed 3× with TBST containing 0.1% BSA, and pellets were resuspended in 200 μL of TBS and then split into two 100 μL aliquots. 50 μL of 50 mM Tris-HCl (pH 7.45) with or without 333 μM of the chromogenic plasmin substrate (H-D-Val-Leu-Lys-pNA) and 50 μL 1.2 μg of tPA or TBS alone was added to each sample and incubated at 37°C for 3 h. Bacteria were pelleted via centrifugation and 150 μL of each supernatant was pipetted into a 96-well plate and absorbance at 405 nm was determined as a measure of plasmin activity.

### Membrane protein fractionation

Outer membrane enriched fractions were isolated by a procedure adapted from de Bruin, et al [53]. FTLVS were grown in BHI broth (500 ml) to mid-log phase and then were pelleted via centrifugation at 6,400 × g for 30 minutes. Cells were resuspended in cold PBS and then lysed by sonication. Unlysed bacterial cells were separated from the whole-cell lysate by centrifugation at 10,000 × g for 20 minutes at 4°C. The insoluble membrane fraction was then isolated by ultracentrifugation for 1 hour at 100,000 × g at 4°C. After removal of the soluble protein fraction, the pelleted total membrane fraction was resuspended in 1% sarkosyl with vortexing and subjected to a second round of ultracentrifugation for 1 hour at 100,000 × g at 4°C. The Sarkosyl-insoluble pellet was resuspended in 50 mM Tris pH 8. The protein concentration of both the Sarkosyl-soluble and Sarkosyl-insoluble fractions was determined using the DC protein assay (Bio-Rad, Hercules, CA) according to manufacturer directions. Samples were stored at -20°C until use.

### Fibronectin degradation assay

Overnight cultures of FTLVS were washed three times with PBS, 10^9 ^CFU were pipetted into 1.5 mL tubes, and bacteria were pelleted via centrifugation at 18,900 × g for 10 minutes. Bacterial pellets were then resuspended in 50 μl of PBS with or without PLG (2 mg/ml), followed by the addition of 50 μl of tPA (10 μg/mL) and incubation at 37°C with gentle shaking for 1 hour. The bacterial suspensions were pelleted via centrifugation at 18,900 × g, washed 3× with PBS and resuspended with 100 μl of 50 mM Tris, 100 mM NaCl, 5 mM CaCl_2_) with 3 μg fibronectin (BD Biosciences) and incubation at 37°C with gentle shaking for 24 hours. After the incubation was complete, bacteria were pelleted via centrifugation at 18,900 × g and the supernatants were solublized by boiling in 2× SDS-PAGE sample buffer containing 2-mercaptoethanol. Samples were subjected to 10% SDS-PAGE and then electrophoretically transferred to a PVDF membrane (Immobilon-P, Millipore). The PVDF membrane was pre-blocked with 1% BSA-TBST for 1 hour at RT to minimize non-specific protein binding, and was then incubated with sheep anti-human fibronectin-specific antibody (diluted 1:2000 in 1% BSA-TBST) for 1 hour at RT with gentle rocking. The PVDF membrane was washed three times with TBST to remove unbound primary antibody. The membrane was then incubated in a solution of anti-sheep/goat IgG monoclonal antibody (GT-34, diluted 1:5000 in 1%BSA-TBST) with rocking for 1 hr at RT. The PVDF membranes were washed 3 times with TBST to remove unbound secondary antibody. The blot was developed using Pierce PicoWest chemiluminescence reagents and images were captured using a Bio-Rad ChemiDoc XRS system.

### Far-Western blotting analysis

Approximately 100 μg of each protein fraction was precipitated using ice-cold acetone, pelleted via centrifugation at 18,900 × g for 15 minutes, and air-dried at room temperature. The samples were then solublized by boiling in 1× SDS-PAGE sample buffer containing 2-mercaptoethanol. Duplicate 20 μL aliquots of each sample were subjected to 15% SDS-PAGE to separate the proteins based on their size. One set of the samples was then electrophoretically transferred to a PVDF membrane (Immobilon-Psq, Millipore). The PVDF membrane was pre-blocked with 1% BSA-TBST for 1 hour at room temperature to minimize non-specific protein binding and was then incubated in a solution of huPLG (3 ug/mL in 1% BSA-TBST) for one hour with rocking at 37°C. Unbound PLG was removed by washing three times with TBST. Sheep anti-human PLG-specific antibody (diluted 1:2,000 in 1% BSA-TBST) was added (100 μL/well) and allowed to incubate for 1 hour at RT° with rocking. The PVDF membrane was washed three times with TBST to remove unbound primary antibody. The membrane was then incubated in a solution of anti-sheep/goat IgG monoclonal antibody (GT-34, diluted 1:5,000 in 1%BSA-TBST) with rocking for 1 hr at room temperature. The PVDF membranes were washed three times with TBST to remove unbound secondary antibody. The blot was developed using Pierce PicoWest chemiluminescence reagents and imaged using a Bio-Rad ChemiDoc XRS system.

### Proteomic identification of PLG-binding FT proteins

Protein bands were excised from Coomassie-stained SDS-PAGE gels, cut into small pieces, incubated in 50% acetonitrile/100 mM ammonium bicarbonate until colorless, and dried via vacuum centrifugation. The protein was digested by adding 20 μl of a 20 ng/μl trypsin solution and incubating overnight at 37°C. Peptides were extracted from the gel slices via sonication in 50 μl 60% acetonitrile/5%TFA, dried via vacuum centrifugation, and reconstituted in 15 μl 0.1% TFA. Tryptic peptides were desalted/enriched using a C18 ZipTip column (Millipore, Billerica, MA) according to manufacturer's instructions and the eluant was spotted on a MALDI plate and dried. Samples were analyzed using a MALDI-LTQ mass spectrometer (ThermoFinnigan, San Jose, CA). A full MS scan in high-mass range (m/z 600-4000, 5 microscans) was performed. The 50 most intense peaks in the full MS spectrum were selected, and MSMS scans were performed for those ions in high-mass range (m/z 50-4000, 5 microscans), the normalized collision energy for MSMS was 35. Xcalibur software was used to process the mass spectrometric data, and the NCBInr database and the Bioworks 3.2 search engine software were used for database searching.

## Authors' contributions

SRC conceived and performed all of the experimental work for the study and drafted the manuscript. JEB, TPH, and MAW both participated in the design of the study and played an important role in drafting the manuscript. MAM participated in the design and coordination of all studies, performed the statistical analyses, and helped to draft the manuscript. All authors read and approved the final manuscript.
